# Supplementary figures and images for: Phylogeographic reconstruction using air transportation data and its application to the 2009 H1N1 influenza A pandemic
Source: PLoS Comput Biol. 2020 Feb 7;16(2):e1007101. doi: 10.1371/journal.pcbi.1007101 (PMC7032730; doi:10.1371/journal.pcbi.1007101)

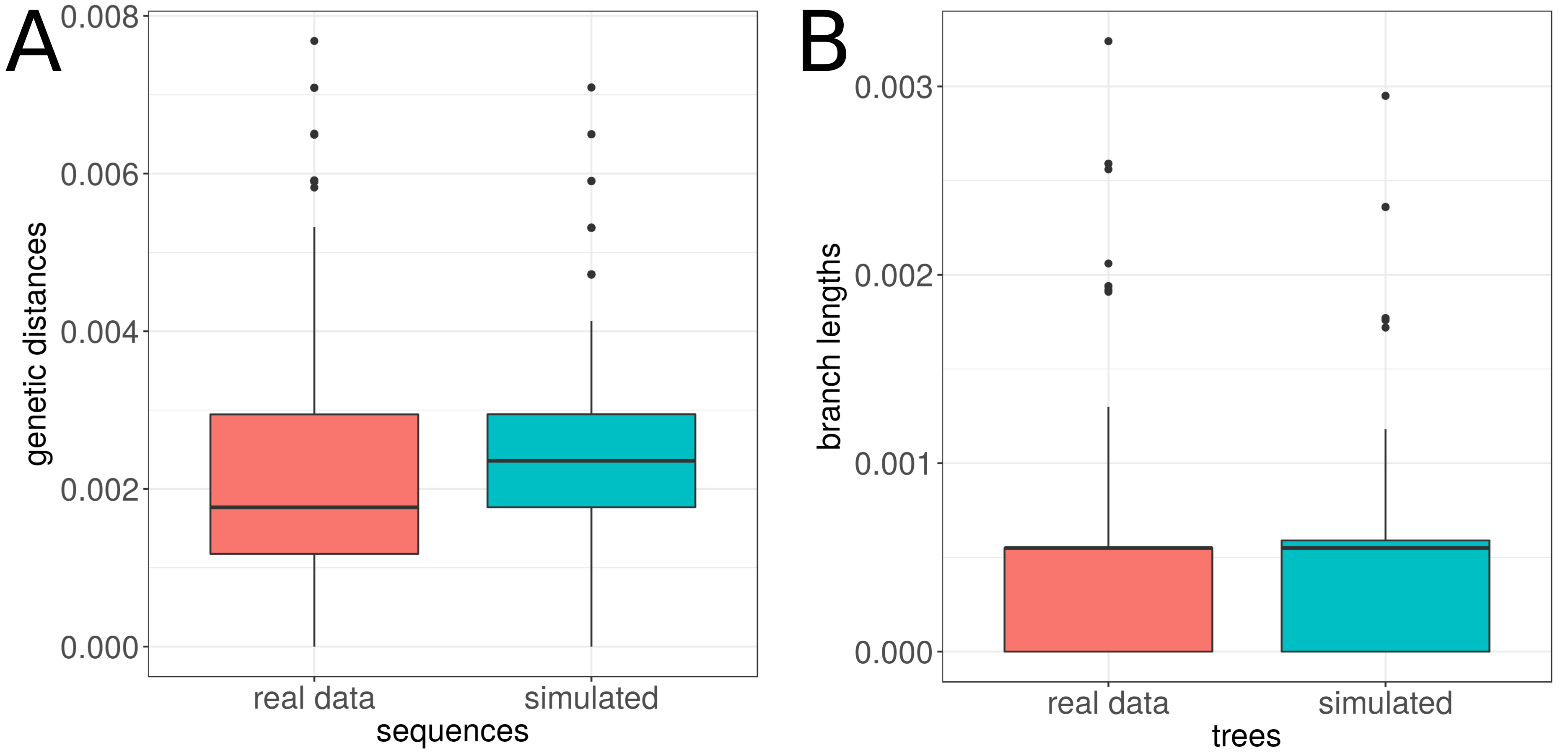

Supplement: S1 Fig — A) Comparison of pairwise genetic distances between sequences for both real HA sequence data and the simulated sequences. B) Comparison of branch lengths on trees inferred on both real HA sequence data and simulated sequences. (PNG) [file pcbi.1007101.s001.png]

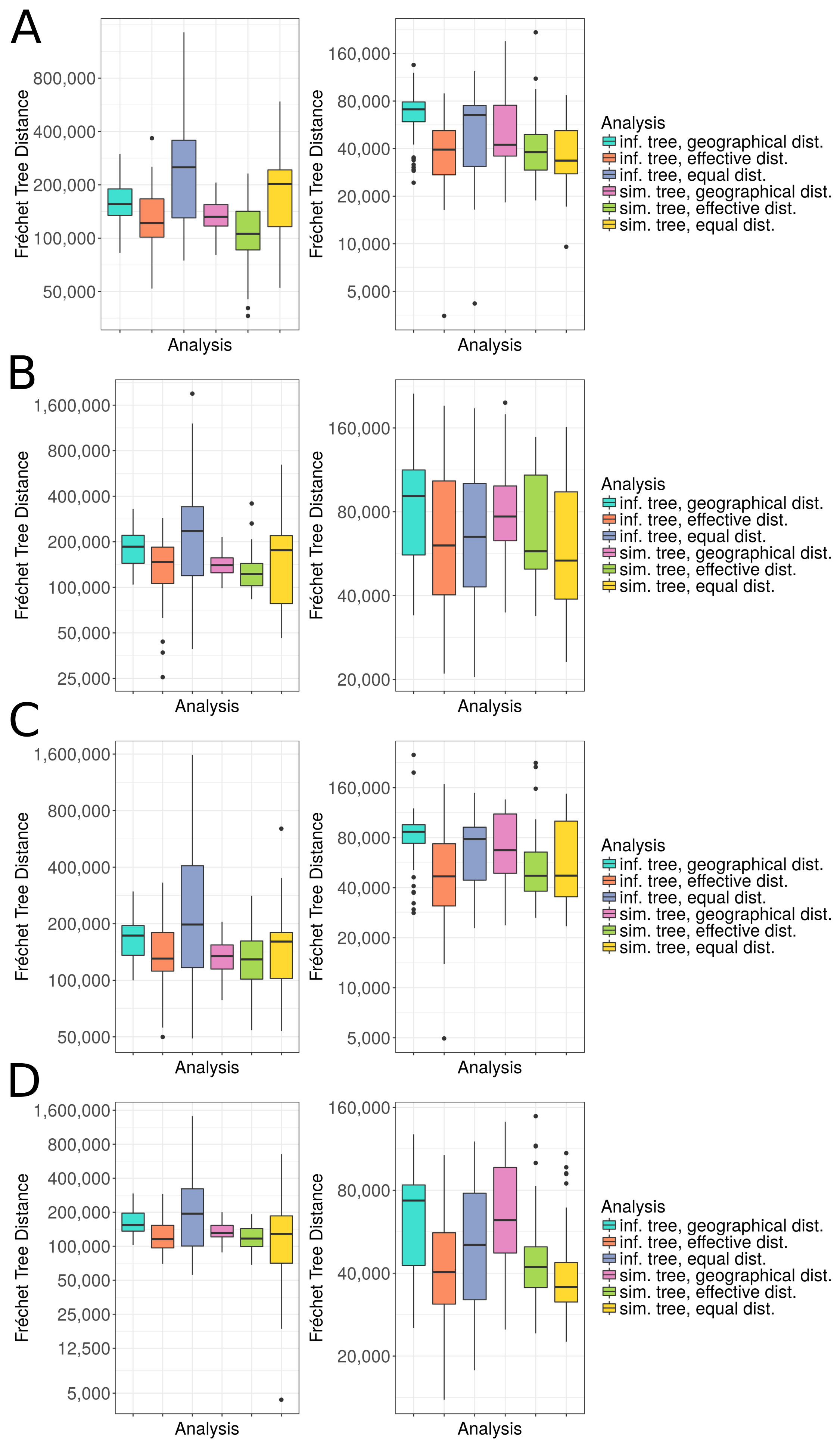

Supplement: S2 Fig — Fréchet tree distances shown on a log2 scale for all six analyzed parsimonious reconstructions using four additional simulations (shown in panels A-D) of geographical spread using GLEAMviz. For each simulation of spread, the sampling, the tree and the sequences were simulated 50 times. Reconstructions were performed both on the airport (on the left) and the country level (on the right). (PNG) [file pcbi.1007101.s002.png]

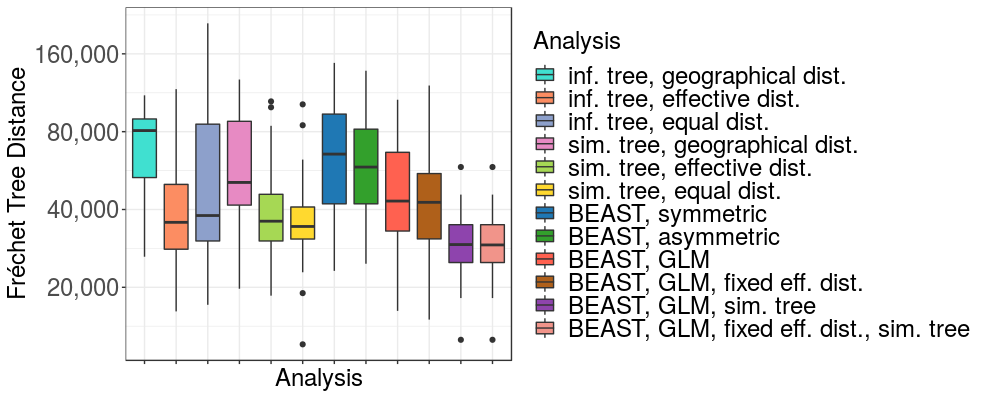

Supplement: S3 Fig — Fréchet tree distances shown on a log2 scale on the country level, including the additional BEAST analyses using the GLM with effective distances as fixed predictors and/or the fixed simulated tree. (PNG) [file pcbi.1007101.s003.png]

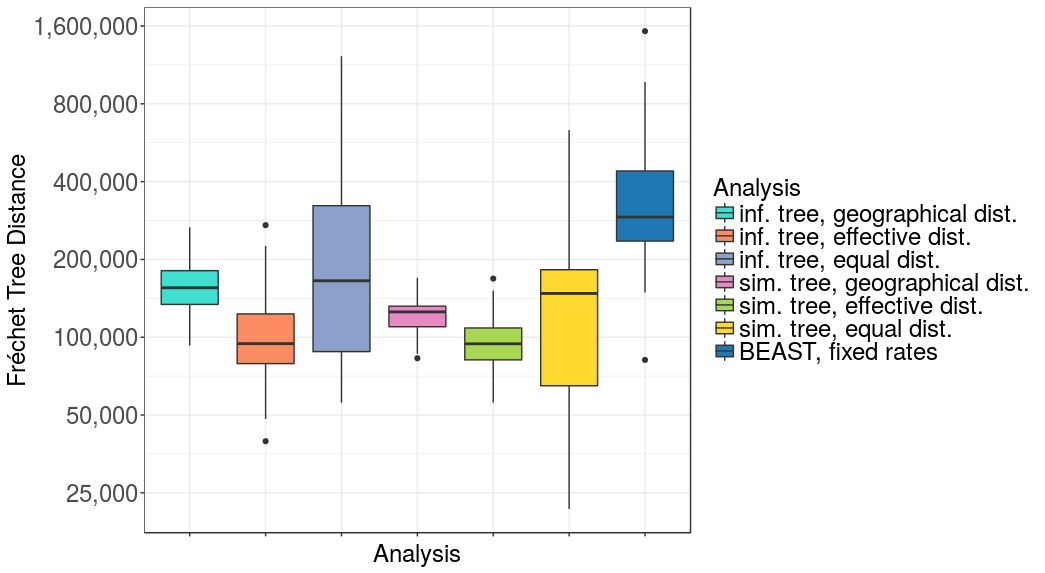

Supplement: S4 Fig — Fréchet tree distances shown on a log2 scale for all six analyzed parsimonious reconstructions on the airport level in comparison to a BEAST reconstruction with fixed rates and a fixed tree topology. The rates were set to inverse effective distances while the tree topology was set to the tree inferred using Fasttree, which allowed for short MCMC runs with 1 million steps and therefore enabled a reconstruction on the airport level with a larger number of locations. (PNG) [file pcbi.1007101.s004.png]
