# Supplementary material for: Phylogeographic reconstruction using air transportation data and its application to the 2009 H1N1 influenza A pandemic
Source: PLoS Comput Biol. 2020 Feb 7;16(2):e1007101. doi: 10.1371/journal.pcbi.1007101 (PMC7032730; doi:10.1371/journal.pcbi.1007101)
Supplement: S1 Table — Main airports involved in the spread of pH1N1, as measured by the number of transitions to new locations. The airports are New York (JFK), Phoenix (PHX), Los Angeles (LAX), Houston (IAH), Mexico City (MEX) and Philadelphia (PHL). The size of the airports in North America was determined by the numbers of passengers in 2013 as given via the OAG database. (PDF) [file pcbi.1007101.s005.pdf]

Airport	Number of transitions	Number of sequences	N-th largest airport in North America
JFK	31	104	5
PHX	24	7	10
LAX	21	29	3
IAH	13	1	11
MEX	11	42	15
PHL	11	4	20

S1 Table: Main airports involved in the spread of pH1N1.
